# A novel combined model integrating collagen properties, radiomics and clinical data to predict gastric cancer prognosis

**DOI:** 10.3389/fonc.2026.1801350

**Published:** 2026-04-10

**Authors:** Yequan Xie, Guangyu Zhong, Jintao Zeng, Zhenqi Meng, Shilin Zhi, Yang Chen, Fanghai Han, Jianan Tan, Shengning Zhou

**Affiliations:** 1 Department of Gastrointestinal Surgery, The Affiliated Guangdong Second Provincial General Hospital of Jinan University, Guangzhou, Guangdong, China; 2 Department of Gastrointestinal Surgery, Sun Yat-Sen Memorial Hospital of Sun Yat-Sen University, Guangzhou, Guangdong, China

**Keywords:** collagen remodeling, CT imaging, gastric cancer, prognostic model, radiomics

## Abstract

**Introduction:**

Gastric cancer (GC) is one of the most common malignant tumors in the world, and there is still no good method to predict the prognosis of GC patients. Collagens are the major component of the extracellular matrix (ECM), and abnormalities in collagen are closely associated with the development of GC.

**Methods:**

This study investigated collagen signatures in GC using bioinformatics analysis and picrosirius red staining. A total of 196 GC patients were included, with 138 assigned to the training group and 58 to the validation group. Multivariate analysis was performed to evaluate prognostic factors, and a predictive model was constructed by integrating collagen properties, radiomics, and clinical data.

**Results:**

The study revealed that collagen signatures were associated with GC initiation and progression. Multivariate analysis confirmed that the picrosirius red risk score (HR = 1.1, 95% CI: 1.02–1.18, p = 0.013) and Radscore (HR = 2.34, 95% CI: 1.3–4.21, p = 0.005) were independent prognostic factors for overall survival. The combined model demonstrated high accuracy, with a C-index of 0.9 in the training group and 0.84 in the validation group, along with good goodness of fit and net benefit.

**Discussion:**

These findings suggest that a prognostic model integrating multiple types of data, including collagen characteristics, radiomics, and clinical factors, can better predict the overall survival of GC patients.

## Background

Gastric cancer (GC) is one of the most common malignant tumors in the world and ranks fourth in malignant tumor-related deaths worldwide (1). In China, GC ranks fifth in incidence and third in deaths among all cancers (2). GC represents an enormous global health burden. Currently, due to the heterogeneity of GC (3), there is still no good method to predict the prognosis of GC patients. Therefore, there is an urgent need to establish an effective prognostic model for GC in order to improve patient survival and quality of life.

The extracellular matrix (ECM) is the structural scaffold of all multicellular organisms (4) and playing a key role in several physiological processes. Collagen is a major component of the ECM and 44 collagen genes have been identified (5, 6). In healthy tissues, collagen production and degradation are in a balance. However, in cancer, the expression of collagen genes is dysregulated, leading to aberrant collagen formation, resulting in loss of tissue structure and cellular behavior, and ultimately disease progression (7, 8).

Abnormalities in collagen are also closely associated with the development of GC. Many studies have shown that the expression of collagen genes (e.g., COL1A1, COL1A2, COL10A1, COL11A1, and COL12A1, etc.) is upregulated in GC tissues, and correlates with progression and poor prognosis of GC (9, 10). High collagen density has also been associated with poor prognosis in GC (11). Therefore, the determination of collagen expression in tissues is very useful in understanding or classifying the pathophysiological conditions of GC (12). Picrosirius red staining is one of the best studied histochemical method to visualise collagen fibres in tissues. It can bind to fibrillar collagens and appears as a bright birefringence signal under polarised light microscopy, while the rest of the tissue remains dark/black. Under light microscopy, all areas of tissue are stained pink by picrosirius red (12, 13). Therefore, a semi-quantitative analysis of the collagen composition can be performed using picrosirius red staining to further investigate the correlation between the degree of collagen enrichment and tumor prognosis. Previous studies have shown that the picrosirius red birefringence signal is significantly associated with survival in lung cancer (14).

Radiology is a non-invasive method used in the diagnosis, treatment monitoring and follow-up of cancer. Radiomics is a high-dimensional, multi-parameter method based on medical images that aims to extract high-throughput data from massive medical images for analysis. By combining machine learning methods with medical images (such as CT, MR, etc.), a large number of features are extracted from the images and converted into quantitative features to be mined (15). The application of radiomics in GC is quite extensive, it can be used alone or in combination with other data (e.g., pathological, clinical, and genomic data) (16) to generate predictive models for immunological characterization (17), therapeutic efficacy (18–20), prognosis (21, 22), metastasis (23, 24), and staging (25) of GC.

However, no study has combined radiomic and collagen features to predict the prognosis of GC. In the present study, we firstly used bioinformatics methods to explore the roles of ECM and collagen in GC development, and then further verified the influence of collagen enrichment on the prognosis of GC using picrosirius red staining. Meanwhile, the clinical value of preoperative enhanced CT images in predicting postoperative survival of GC patients was investigated using radiomics methods. Finally, a novel model for predicting GC survival outcome was constructed by integrating picrosirius red risk score, radiomics and clinical data. It may provide a basis for predicting the prognosis of GC and constructing individualised follow-up and treatment strategies.

## Methods

### Data sources

High-throughput RNA sequencing data from 34 normal gastric tissues and 379 gastric cancer (GC) tissues were downloaded from The Cancer Genome Atlas (TCGA) database (TCGA-STAD cohort; https://portal.gdc.cancer.gov/). To ensure comparability with microarray data, these transcriptomic profiles were normalized using Transcripts Per Million (TPM) values. Clinical information for the TCGA-STAD cohort was retrieved via the TCGAbiolinks package for subsequent analysis. Additionally, microarray datasets comprising 1,177 GC patients were obtained from the Gene Expression Omnibus (GEO) database, including Affymetrix platform datasets (GSE62254, GSE15459) and Illumina platform datasets (GSE26899, GSE26901, GSE84437; https://www.ncbi.nlm.nih.gov/geo/). Corresponding clinical information for these GEO cohorts was either downloaded directly from the GEO database or extracted from the **Supplementary Materials** of the original publications.

GC tissue from primary GC were obtained from 196 patients who underline surgery resection between 2015 and 2020 at Sun Yat-sen Memorial Hospital, Guangzhou, China. Patients’ inclusion criteria: (1) clinical information was well established, (2) preoperative CT imaging was adequately documented, and the quality of the CT images was sufficient to meet the clinical requirements, (3) underwent for laparoscopic gastrectomy with D2 lymphadenectomy, (4) histologically proven gastric adenocarcinoma, and the TNM staging is well defined, (5) postoperative survival time exceeded three months. Exclusion criteria: (1) incomplete clinical, imaging or pathology data, (2) history of upper abdominal surgery, (3) history of neoadjuvant therapy, (4) under emergency surgery. The patients’ baseline is included in the supplementary file. All patients had signed written informed consent for usage their biology specimen in the studies. The study was approved by Sun Yat-sen Memorial Hospital Ethics Committee, in agreement with the Helsinki declaration.

### Bulk RNAseq analysis and pathway enrichment analyses

For the TCGA data, genes were filtered when a gene count was expressed in less than twenty percent samples. Read counts were TMM normalized using edgeR package. Normalized RNAseq abundances were log2 transformed. Differential gene expression analysis was performed by the voom algorithm (default settings, limma package). LmFit algorithm was used for fitting data using a linear model (trend = TRUE, limma package). Differential expression between different group was calculated by eBayes algorithm (default settings, limma package). The DE collagen genes were defined as the intersection of DE genes and collagen genes list according to Naba et al (5, 26). Heatmaps of gene expression were generated using the ComplexHeatmap package. For the GEO datasets, the microarray datasets from the same platform were integrated together for further analysis and a batch normalization algorithm (sva package) was employed to remove batch effects.

The pathway enrichment analysis was performed on the 50 hallmark pathways and the canonical pathways derived from Reactome pathway database using the Msigdb database(https://www.gsea-msigdb.org/gsea/msigdb). The human gene information was obtained from ‘org.Hs.eg.db’ package. The gene symbol used for enrichment analysis was transformed into Entrez ID using bitr algorithm (OrgDb = org.Hs.eg.db, ClusterProfiler package).

The enricher algorithm (default setting, ClusterProfiler package) was used for enrichment. If p < 0.05, the pathway was considered to be significantly enriched. ‘ggplot2’ package, ‘ggpubr’ package were used for visualization.

### Collagen subtype identification

To identify the GC subtypes, Monte Carlo reference-based consensus clustering (M3C algorithm, clusteralg=c(‘pam’), M3C package) was applied to the expression matrix of DE collagen genes from TCGA-STAD tumor compared to normal tissue. Optimal cluster number was determined according to a maximal Relative Cluster Stability Index, a Monte-Carlo *p*-value less than 0.1 and a minimal Proportional of Ambiguous Clustering Score (27).

The mean Z-scaled expression level for DE collagen genes from the gastric tumor compared to normal tissue was used to calculate the centroids of the TCGA GC collagen subtypes. For GEO datasets, Euclidean distance would be calculated between each sample and the centroids of each collagen subtype. Each sample would be assigned to one of the collagen subtypes according to the smaller distance.

### Picrosirius red staining and quantitation in tissue

Picrosirius red staining of formalin-fixed, paraffin-embedded GC tissue sections was performed as previously described (28). Paraffin-embedded tissue microarrays were cut into 4 μm sections. The sections were warmed on a hot plate at 60 °C for 3 hours and then deparaffinized in xylene three times (5 min each). The slides were subsequently rehydrated through a graded ethanol series (100%, 95%, and 75%; 2 min each). The paraffin sections were then stained with hematoxylin for 5–10 min to visualize nuclei and washed under running tap water for 2 min. Afterwards, the sections were stained with 0.1% picrosirius red for 1 hour to detect fibrillar collagen. The slides were rinsed with freshly prepared acidified water and dehydrated through a graded ethanol series (75%, 95%, and 100%). Finally, the sections were cleared in xylene three times (5 min each) and mounted with a resinous medium. The stained sections were imaged using a bright-field microscope (Leica DMI) and a polarization microscope (Leica DMI 6000). The picrosirius red signal was quantified using ImageJ software (National Institutes of Health, Bethesda, MD, USA; version 1.53). The picrosirius red risk score was defined as the total red-stained area normalized to the total tissue area. For each patient, the average picrosirius red risk score from three to five cores was calculated. Based on the mean value of the picrosirius red risk score, patients were classified into high-risk and low-risk groups.

### Radiomic study

For the radiomics analysis, the entire cohort was randomly divided into a training set (n = 138) and a validation set (n = 58) at a ratio of 7:3 using the createDataPartition function in the caret package. Baseline clinical characteristics were compared between the two cohorts to ensure comparability. All patients underwent contrast-enhanced CT using a Siemens SOMATOM Sensation 64 scanner. Patients fasted for at least 6 hours prior to examination and were instructed to drink 500–1000 mL of water before scanning to achieve gastric distension. Scanning was performed in the supine position from the diaphragm to the pubic symphysis. The scanning parameters were as follows: tube voltage 120 kV, automatic tube current modulation, slice thickness and spacing of 5 mm, reconstructed slice thickness and spacing of 1.25 mm, matrix size 512 × 512, and field of view 350 mm × 350 mm. Contrast medium (iodixanol, Omnipaque 300; GE Healthcare, Chicago, IL, USA; 1.2 mL/kg) was injected through the right antecubital vein at a rate of 2.5 mL/s using a high-pressure injector, followed by a 20 mL saline flush. Arterial phase images were acquired 20 s after triggering when the attenuation of the descending aorta reached 100 HU, and portal venous phase images were obtained after a delay of 60 s. Patients were instructed to hold their breath during image acquisition to reduce motion artifacts. Tumor segmentation was performed using 3D Slicer software (version 5.5.0). ROIs were manually delineated along the tumor boundaries on arterial phase images (**Supplementary Figure 1**), as features derived from arterial phase images have been reported to demonstrate superior predictive performance compared with portal or venous phases (29). The ROI delineation was independently performed by two radiologists with more than five years of experience. Areas of bleeding, necrosis, and indistinct tumor margins were carefully avoided.

Radiomics features were extracted using Python (version 3.6.13). A total of 1,037 radiomic features were extracted from each ROI, including 107 original features, 186 Laplacian of Gaussian features, and 744 wavelet features. Feature normalization was performed using Z-score normalization based on the training cohort. The mean and standard deviation derived from the training cohort were subsequently applied to standardize the radiomic features in the validation cohort. To assess the reproducibility of feature extraction, 40 cases were randomly selected and the ROIs were re-delineated by the two radiologists after an interval of four weeks. Intra- and inter-observer reproducibility were evaluated using the intraclass correlation coefficient (ICC). Radiomic features with ICC ≥ 0.75 were considered to have good reproducibility and were retained for further analysis. For radiomic model construction, univariate Cox regression analysis was first performed to identify features significantly associated with survival (p < 0.01). Subsequently, the least absolute shrinkage and selection operator (LASSO) regression method with 10-fold cross-validation was applied to select the optimal penalty parameter (λ). Radiomic features with non-zero coefficients were finally retained and linearly combined to generate the radiomics score (Radscore).

### The construction of prediction model

We used univariate Cox regression analysis to preliminarily screen clinical features related to GC survival outcomes from the training group (p<0.05). Among them, we transformed serum tumor markers into binary variables. The critical values for CEA, AFP, CA125, CA19-9, and CA72–4 are 5 ng/mL, 7 ng/mL, 25 U/ml, 34 U/ml, and 7 U/ml, respectively, which were determined by the testing center of Sun Yat Sen Memorial Hospital, taking into account both environmental and patient-specific factors. To further determine the degree of collinearity between the variables, the variance inflation factor (VIF) method (car package, “VIF” algorithm) was used to perform collinearity diagnosis on the independent variables that were obtained from univariate Cox regression. The variables with VIF>10 were removed. The non-collinear features were then used in a multivariate Cox regression analysis to further identify independent risk factors (p<0.05) affecting GC survival outcomes from the training group data. Then we used the independent risk factors to construct the Cox proportional hazards regression model to predict the survival outcome of GC. Both univariate and multivariate Cox regression analyses were performed using the “coxph” algorithm (survival package (30)). Meanwhile, the hazard ratio of risk factors affecting GC survival outcomes were calculated.

### The model evaluation and the construction of nomogram

We evaluate the predictive performance of the model using the consistency index (C-index), which ranges from 0.5 to 1, with higher values indicating better predictive performance. The “timeROC” algorithm (timeROC package) was used to plot the receiver operating characteristic(ROC) curve and calculate the area under the curve (AUC) to evaluate the diagnostic performance of the model. RMS package was used to draw calibration curves and evaluate the goodness of fit of the model. The “brier_score” algorithm (survey package) was used to calculate the Brier Score (BS) to evaluate the accuracy of the model. The “DCA” algorithm (ggDCA package) was used to draw decision curves to measure the clinical practicality of the model. For the convenience of the clinical application, we use the “nomogram” algorithm (RMS package) to construct a nomogram for the visualization of the combined model.

### Statistics

All statistical analysis was performed in R (v4.2.2). The Kolmogorov-Smirnov test was used to test the normal distribution of quantitative data, while non-normal quantitative data were represented by the median (M [P25, P75]). The Wilcoxon test was used to compare two groups and the Kruskal-Wallis test was used for more than two groups. Comparison of count data between groups was performed using the Chi-square test. OS time and RFS time are measured in years or months, with the patient’s survival status (relapse or death) as the outcome variable. The Kaplan–Meier plotter was used to plot the prognostic survival curve for different groups(“survfit” algorithm, survminer package), and the log-rank test was used for the evaluation of survival differences between groups with statistical significance. Univariate and multivariate Cox regression analysis was performed to calculate the hazard ratio of risk factors for recurrence and death using “coxph” algorithm (survival package). All the tests were conducted at a significance level of 95% and *p*-values less than 0.05 were considered statistically significant.

## Results

### Collagen remodeling promotes GC occurrence

**Figure 1:**
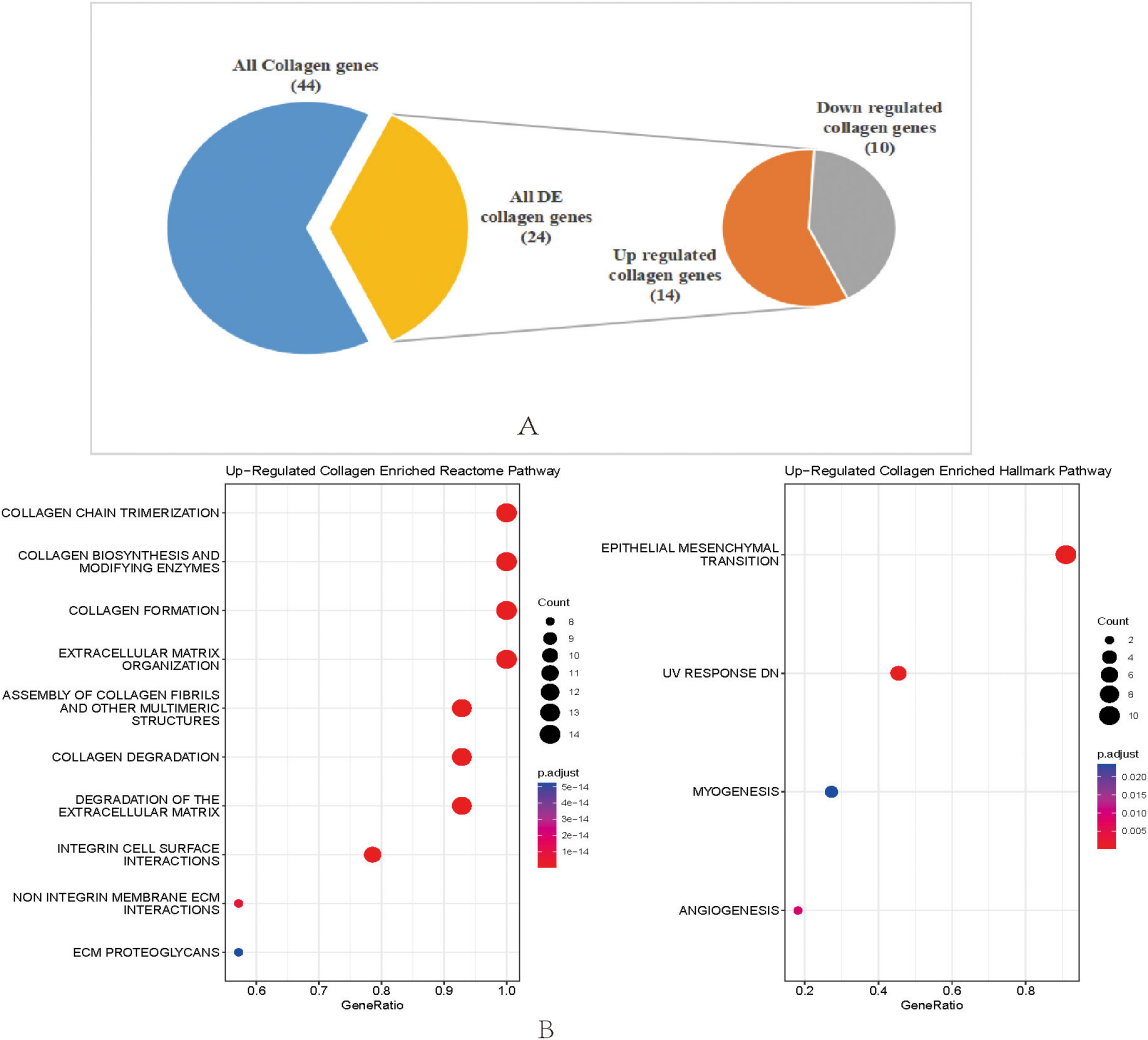
**(A)** Distribution of differentially expressed collagen genes between tumor and non-tumor. 14 collagen genes were up-regulated in GC, while 10 collagen genes were down-regulated in GC. **(B)** Enrichment analysis for the up-regulated collagen genes in GC, left figure represented the MSigDb Reactome pathways and right figure represented the MSigDb hallmark pathways.

In order to investigate the effect of collagen on GC occurrence, we used high throughput RNA sequencing data from 379 gastric adenocarcinoma tissue and matched 34 normal tissues in The Cancer Genome Atlas (TCGA) database for analysis. Differential gene expression analysis between tumoral and normal gastric tissue revealed a total of 24 differentially expressed (DE) collagen genes (**Figure 1A**). Most collagen genes were included. Compared to normal tissue, 14 collagen genes were up-regulated (COL10A1, COL11A1, COL12A1, COL1A1, COL1A2, COL3A1, COL4A1, COL5A1, COL5A2, COL6A3, COL7A1, COL8A1, COL9A1, COL22A1) and 10 collagen genes were down-regulated (COL21A1, COL25A1, COL14A1, COL17A1, COL19A1, COL2A1, COL4A5, COL4A6, COL23A1, COL6A5) in gastric tumor tissue respectively (**Figure 1A**). We used the up-regulated collagen genes in gastric tumor tissue to perform enrichment analysis using the MSigDb pathway database. The results showed these genes were enriched in the formation and degradation of collagen and collagen-integrin receptor interaction of Reactome pathway and epithelial mesenchymal transition (EMT), myogenesis, angiogenesis of Hallmark pathway (**Figure 1B**). Taken together, these results demonstrate a high correlation between collagen remodeling and the GC occurrence.

### Collagen remodeling contributes to GC development

**Figure 2:**
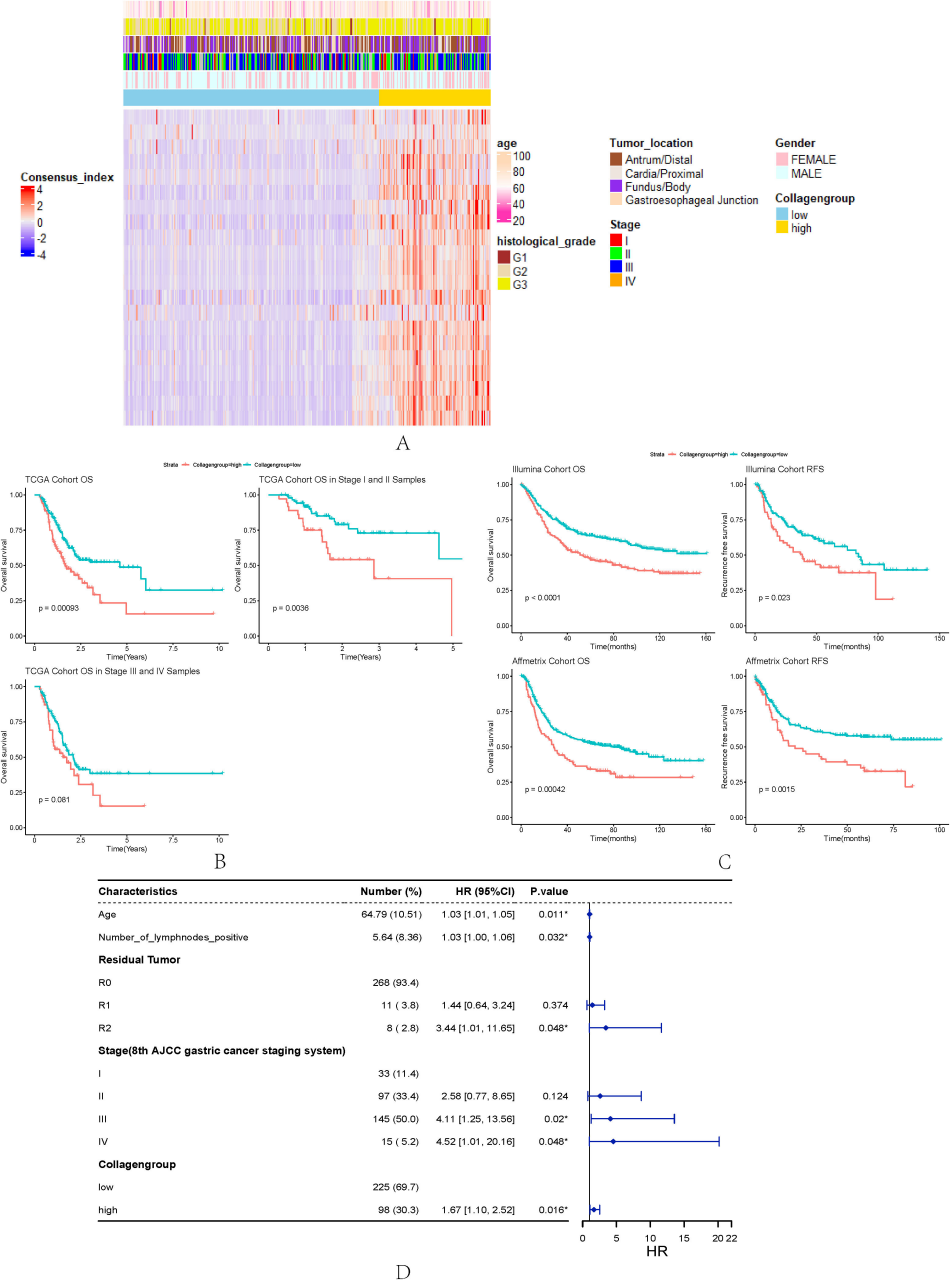
**(A)** Heatmap of collagen genes for the Collagen-High and Collagen-Low group in the TCGA cohort. **(B)** OS analysis between different collagen subtypes in all stage, early stage, and late stage in the TCGA cohort. **(C)** OS and RFS analysis between different collagen subtypes in all stage in the Affymetrix platform cohort and illumina platform cohort. **(D)** Forest plot for multivariate Cox regression analysis of collagen subtype and clinical characteristics for TCGA cohort. *p < 0.05.

Next, we investigate how collagen remodelling affects GC progression. We used the expression of DE collagen genes between GC and normal gastric tissue to classify GC into different groups. Unsupervised clustering of DE collagen genes divided GC samples into two clusters according to best K-value (Method, **Supplementary Figure 2**). One cluster showed high expression of collagen genes (Collagen-High), while the other exhibited low expression (Collagen-Low) (**Figure 2A**). Kaplan–Meier analysis showed that the overall survival (OS) of Collagen-High group was significantly worse than that of Collagen-Low group (**Figure 2B**). The same trend of poor survival was also observed in both the early (stage I and II) and late (stage III and IV) stage GC patients (**Figure 2B**). Then, we used the GEO Affymetrix platform dataset and the Illumina platform dataset to externally validate the prognostic association of collagen groups (Method). As the results showed, whether in the Affymetrix platform or illumina platform datasets, Collagen-High group had worse OS than patients in the Collagen-Low group. The recurrence-free survival (RFS) displayed a similar trend (**Figure 2C**). To evaluate the independent factors affecting the prognosis of GC patients, we performed univariate and multivariate Cox regression analyses. Our results showed that age, the number of positive lymph nodes, R2 residual tumor, stage, and the Collagen-High group were significant independent factors affecting GC prognosis (**Figure 2D**).

We do the differential gene expression analysis between two collagen groups. The volcano plot (**Supplementary Figure 3A**) illustrates the DE genes between the two subtyes (|logFC|> 1 and p.adj < 0.05). We found 21 collagen genes that were highly expressed in the Collagen-High group. Among them, type I collagen genes were the most highly up-regulated collagen genes for the Collagen-High group (including COL3A1, COL8A1, COL10A1). 10 collagen genes were upregulated in both tumor tissue and Collagen-High group (**Supplementary Figure 3B**), suggesting that these genes may be associated with the occurrence and progression of GC.

### Picrosirius red staining predicts the prognosis of GC

**Figure 3:**
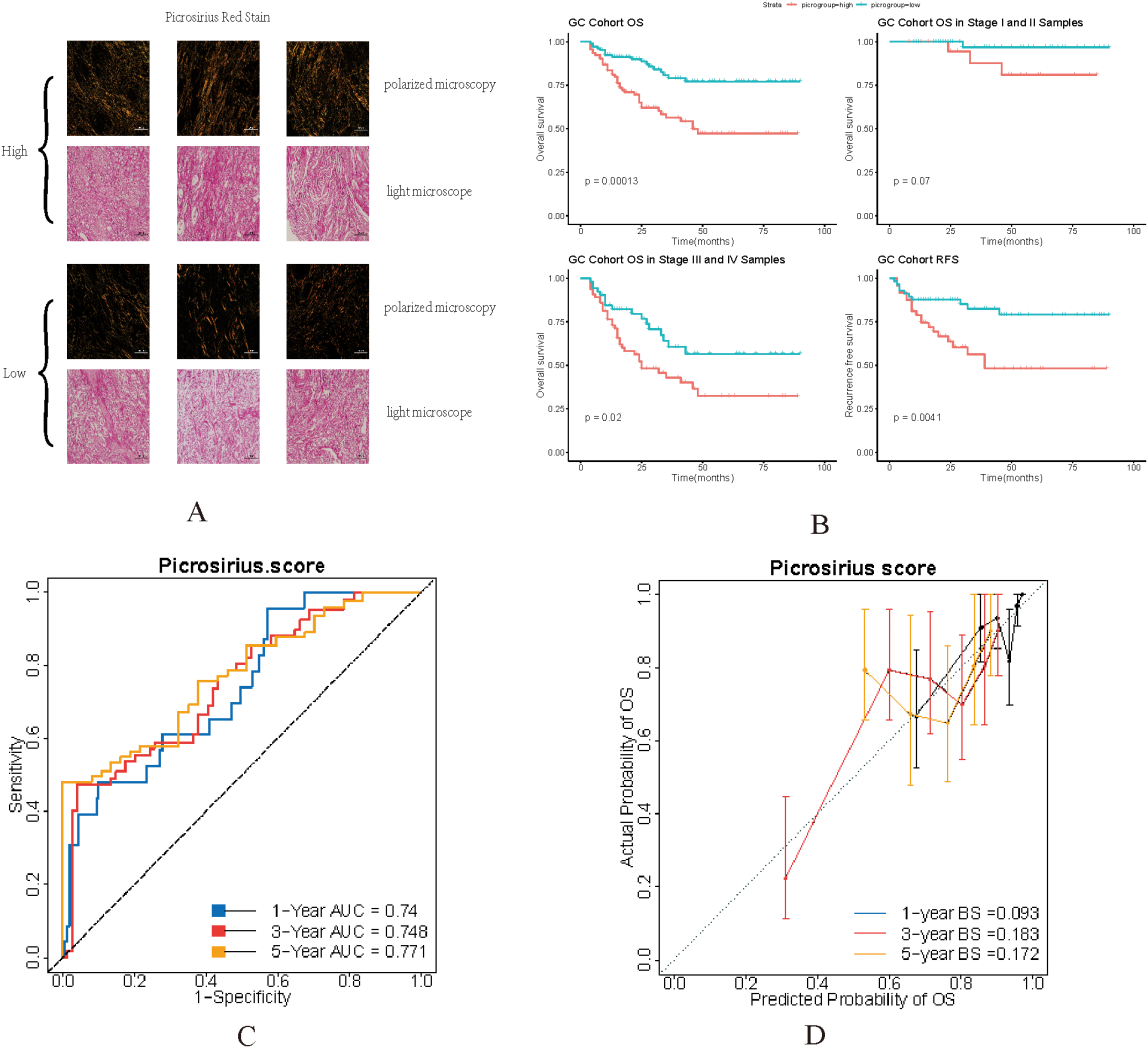
**(A)** representative tissue microarray cores stained with picrosirius red were examined under polarized and light microscopy. The samples corresponded to high and low levels of picrosirius red staining in GC. Scale bar = 100 µm. **(B)** OS and RFS analysis between different picosirius red staining group for GC in our cohort. **(C)** ROC curve for 1, 3, 5 year OS prediction of GC patients with picrosirius red risk score. **(D)** Calibration curve for 1, 3, 5 year OS prediction of GC patients with picrosirius red risk score.

Based on previous results, the expression of collagen genes which significantly associated with collagen subtypes can predict the prognosis of GC patients. Next, we used the birefringence signal of picrosirius red staining under polarized microscopy to represent the amount of fibrillar collagens. The strength of the birefringence signal was calculated as picrosirius red risk score. According to the average value of picrosirius red risk score(14.15695), patients were divided into high picrosirius red staining group and low picrosirius red staining group (**Figure 3A**, Method).

As the result shown, high picrosirius red strength was significantly associated with poor OS and RFS in GC patients, especially in late stage patients (**Figure 3B**). **Supplementary Table 1** shows the differences in baseline data of GC patients under different picrosirius red staining subgroups. It can be seen that in the high picrosirius red staining group, the proportion of stage III (56%) and stage IV (8%) GC patients is higher (p=0.038), the number of positive lymph nodes is higher (7.5 vs 2, p=0.001), and the postoperative mortality rate is significantly higher (41.3% vs 17.3%, p<0.001) than low picrosirius red staining group, which is consistent with the previous results (31, 32).

The results of univariate Cox regression (HR = 1.22, 95%CI: 1.14~1.31, p<0.001, **Table 1**) and multivariate Cox regression analysis (HR = 1.1, 95%CI: 1.02~1.18, p=0.013, **Table 2**) also showed that picrosirius red risk score was the independent factor influencing GC prognosis. Then, we further analyzed the diagnostic efficacy of the picrosirius red risk score in predicting OS of GC patients. The C-index of picrosirius red risk score for predicting OS in GC patients is 0.736. As shown in **Figure 3C**, the AUC values of the ROC curves for predicting 1, 3, 5 year OS of GC patients using the picrosirius red risk score are 0.74, 0.748, and 0.771, respectively. **Figure 3D** shows the calibration curve of picrosirius red risk score for predicting OS in GC patients. The results showed a high consistency between the predicted 1, 3, 5 year OS of GC patients using the picrosirius red risk score and the actual results.

**Table 1:** Univariate Cox regression analysis for predicting the prognosis of GC patients.

Factor	HR	Lower.95	Upper.95	P
Sex	0.72	0.39	1.34	0.305
Age	1.01	0.99	1.04	0.395
height	0.97	0.94	1.01	0.146
weight	0.95	0.92	0.98	0.001
BMI	0.8	0.71	0.91	0.001
History of smoking	0.73	0.38	1.39	0.341
Tumor location	0.98	0.76	1.26	0.854
Surgery method	0.97	0.72	1.31	0.863
Operation time	1	1	1.01	0.262
Blood loss	1	1	1	0.867
Number of lymph nodes	1.04	1.02	1.06	<0.001
Picrosirius red risk score	1.22	1.14	1.31	<0.001
TNM stage	4.14	2.49	6.89	<0.001
T	4.67	2.55	8.54	<0.001
N	2.04	1.56	2.67	<0.001
M	5.1	2.12	12.25	<0.001
Her.2	0.68	0.46	1.01	0.055
Pathology	4.19	1.64	10.68	0.003
Chemotherapy	8.90	2.14	37.01	0.003
Postoperative hospital days	1.04	1	1.08	0.071
Preoperative leukocyte	0.94	0.8	1.11	0.481
Preoperative neutrophil	1.01	0.88	1.17	0.877
Preoperative lymphocyte	0.57	0.34	0.94	0.028
Preoperative hemoglobin	0.99	0.98	1	0.229
Preoperative platelet	1	1	1	0.071
Preoperative neutrophil to lymphocyte ratio	1	0.92	1.08	0.94
Preoperative platelet to lymphocyte ratio	1	1	1	0.107
Preoperative albumin	0.96	0.89	1.03	0.228
Preoperative globulin	1.04	0.98	1.11	0.149
Preoperative total bilirubin	0.96	0.89	1.03	0.248
Preoperative ALT	0.96	0.92	1.01	0.091
Preoperative AST	0.99	0.94	1.05	0.723
Preoperative urea	1.04	0.87	1.25	0.659
Preoperative creatinine	1	0.99	1.02	0.581
Preoperative glucose	1.2	0.97	1.5	0.1
Preoperative AFP	5.31	1.22	23.03	0.026
Preoperative CEA	1.84	0.9	3.75	0.095
Preoperative CA125	5.93	2.41	14.62	<0.001
Preoperative CA19-9	1.67	0.74	3.77	0.218
Preoperative CA72-4	3.51	1.85	6.64	<0.001
Postoperative leukocyte	1.02	0.92	1.13	0.696
Postoperative neutrophil	1.05	0.94	1.16	0.417
Postoperative lymphocyte	0.62	0.31	1.24	0.174
Postoperative hemoglobin	0.98	0.96	1	0.019
Postoperative platelet	1	1	1	0.239
Postoperative neutrophil to lymphocyte ratio	1.05	1	1.1	0.041
Postoperative platelet to lymphocyte ratio	1	1	1	0.038
Postoperative albumin	0.95	0.88	1.02	0.153
Postoperative globulin	1.06	0.98	1.14	0.128
Postoperative total bilirubin	1	0.97	1.04	0.911
Postoperative ALT	0.99	0.98	1	0.223
Postoperative AST	1	0.99	1.01	0.918
Postoperative urea	1.04	0.92	1.18	0.504
Postoperative creatinine	1	0.99	1.02	0.46
Postoperative glucose	0.96	0.82	1.12	0.6
Radscore	5.60	3.60	8.71	<0.001

**Table 2:** Multivariate Cox regression analysis and combined model for predicting the prognosis of GC patients.

Factor	β	HR	Lower.95	Upper.95	P
BMI	-0.16	0.85	0.75	0.97	0.012
Picrosirius red risk score	0.09	1.1	1.02	1.18	0.013
T stage	0.99	2.69	1.46	4.92	0.001
preoperative CA125	1.25	3.48	1.14	10.62	0.028
Radscore	0.85	2.34	1.3	4.21	0.005

Together, these data demonstrated the value of collagen gene expression and the picrosirius red staining for predicting the prognosis of GC patients.

### The extraction of radiomic features and the construction of Radscore

To construct the Radscore, we divided the patients into training group and validation group. Among them, 138 cases were enrolled in the training group and 58 cases were enrolled in the validation group. There were no significant differences in the patient baseline data between them (p>0.05), which indicated the adequacy of the randomization (**Supplementary Table 2**).

After grouping, we used 3D Slicer to delineate the ROI of the enhanced CT arterial phase images of the enrolled GC patients. A total of 1037 radiomics features were extracted from the images using Python. Among them, 872 radiomics features were considered stable features after ICC evaluation.Through univariate Cox regression analysis, a total of 564 radiomics features were found to be significantly correlated with the prognosis of GC patients in the training group (p<0.01).

**Figure 4:**
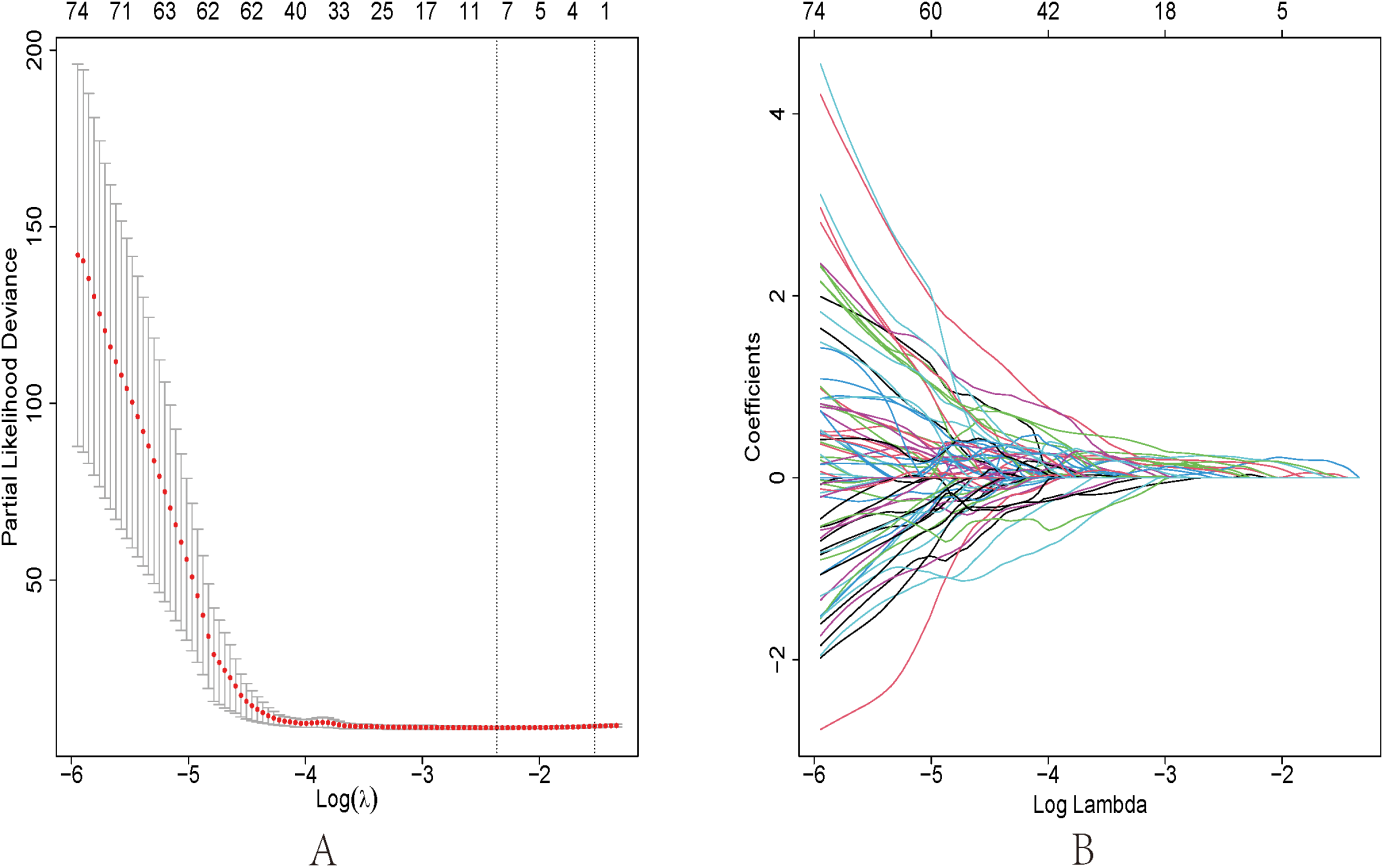
**(A)** LASSO regression 10-fold cross-validation curve. λ was selected when the mean square error was minimized. **(B)** LASSO regression coefficient path diagram using 10-fold cross-validation.

Then, we used LASSO regression to further screen these 612 features and performed 10-fold cross-validation to select the optimal lambda value. When λ=0.0940405, the mean square error was minimized (**Figures 4A, B**). And a total of 11 radiomics features with non-zero coefficients were obtained (**Table 3**). It included 3 original features, 5 Laplacian feature, and 3 wavelets features. Among them, log.sigma. 3mm. 3D_glszm_SizeZoneNormality has the highest coefficient. It belongs to Gray Level Size Zone Matrix (GLSZM) Features. Therefore, the formula for Radscore is equal to the following::original_shape_Maximum2DDiameterRow×0.029784856+original_firstorder_Range×0.016033553+original_glcm_ClusterProminence×0.057746809+log.sigma.3.mm.3D_firstorder_Maximum×0.104282567+log.sigma.3.mm.3D_glrlm_RunLengthNonUniformity×0.138738029+log.sigma.3.mm.3D_glszm_SizeZoneNonUniformity×0.224102528+log.sigma.3.mm.3D_ngtdm_Busyness×0.019902341+log.sigma.3.mm.3D_ngtdm_Complexity×0.025613263+wavelet.LLH_firstorder_Kurtosis×0.170823405+wavelet.LLH_glszm_LargeAreaEmphasis×0.12952544+wavelet.LLL_firstorder_Kurtosis×0.194280494.

**Table 3:** Screening results of radiomics features.

Radiomics feature	Coefficient
original_shape_Maximum2DDiameterRow	0.029784856
original_firstorder_Range	0.016033553
original_glcm_ClusterProminence	0.057746809
log.sigma.3.mm.3D_firstorder_Maximum	0.104282567
log.sigma.3.mm.3D_glrlm_RunLengthNonUniformity	0.138738029
log.sigma.3.mm.3D_glszm_SizeZoneNonUniformity	0.224102528
log.sigma.3.mm.3D_ngtdm_Busyness	0.019902341
log.sigma.3.mm.3D_ngtdm_Complexity	0.025613263
wavelet.LLH_firstorder_Kurtosis	0.170823405
wavelet.LLH_glszm_LargeAreaEmphasis	0.12952544
wavelet.LLL_firstorder_Kurtosis	0.194280494

### The evaluation of Radscore in predicting the prognosis of GC patients

**Figure 5:**
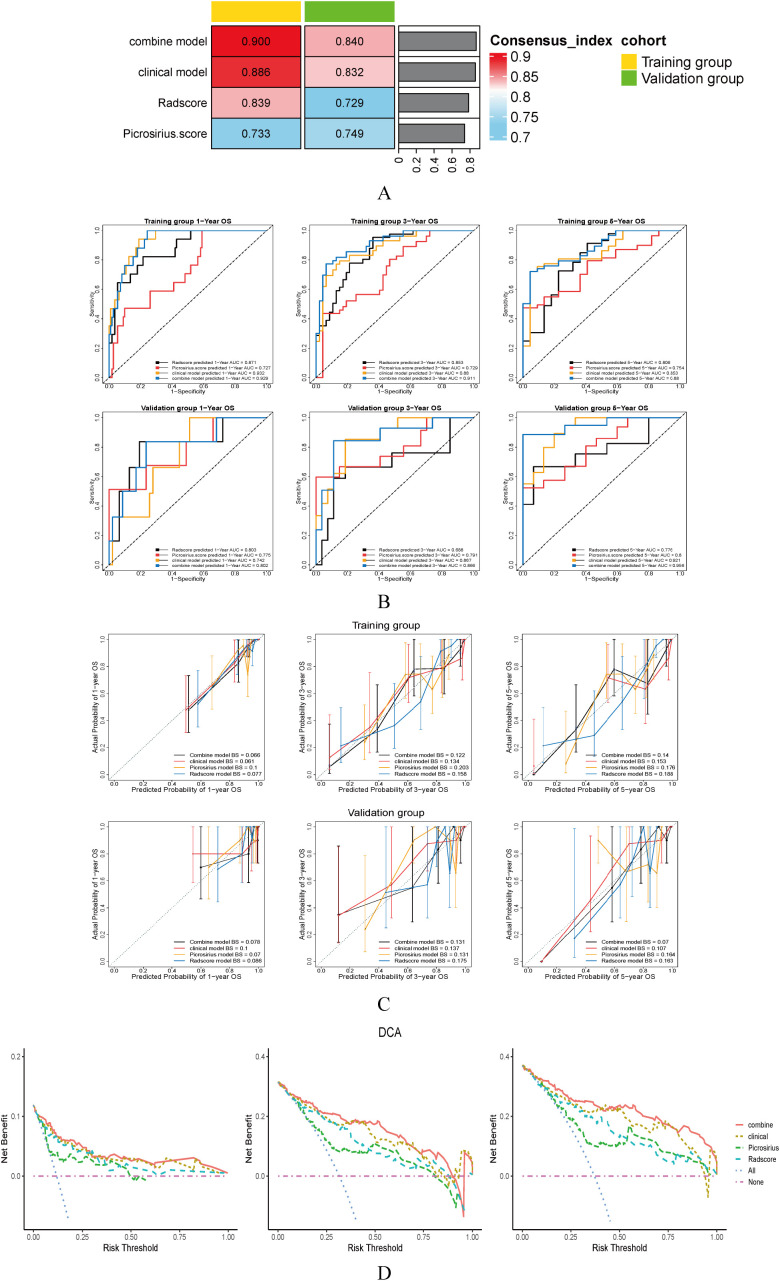
**(A)** C-index of each model in training and validation group. **(B)** ROC curve of for 1, 3, 5-year OS prediction of GC patients with each model. **(C)** Calibration curve for 1, 3, 5 year OS prediction of GC patients with each model. **(D)** Decision curve for 1, 3, 5 year OS prediction of GC patients with each model.

**Figure 6:**
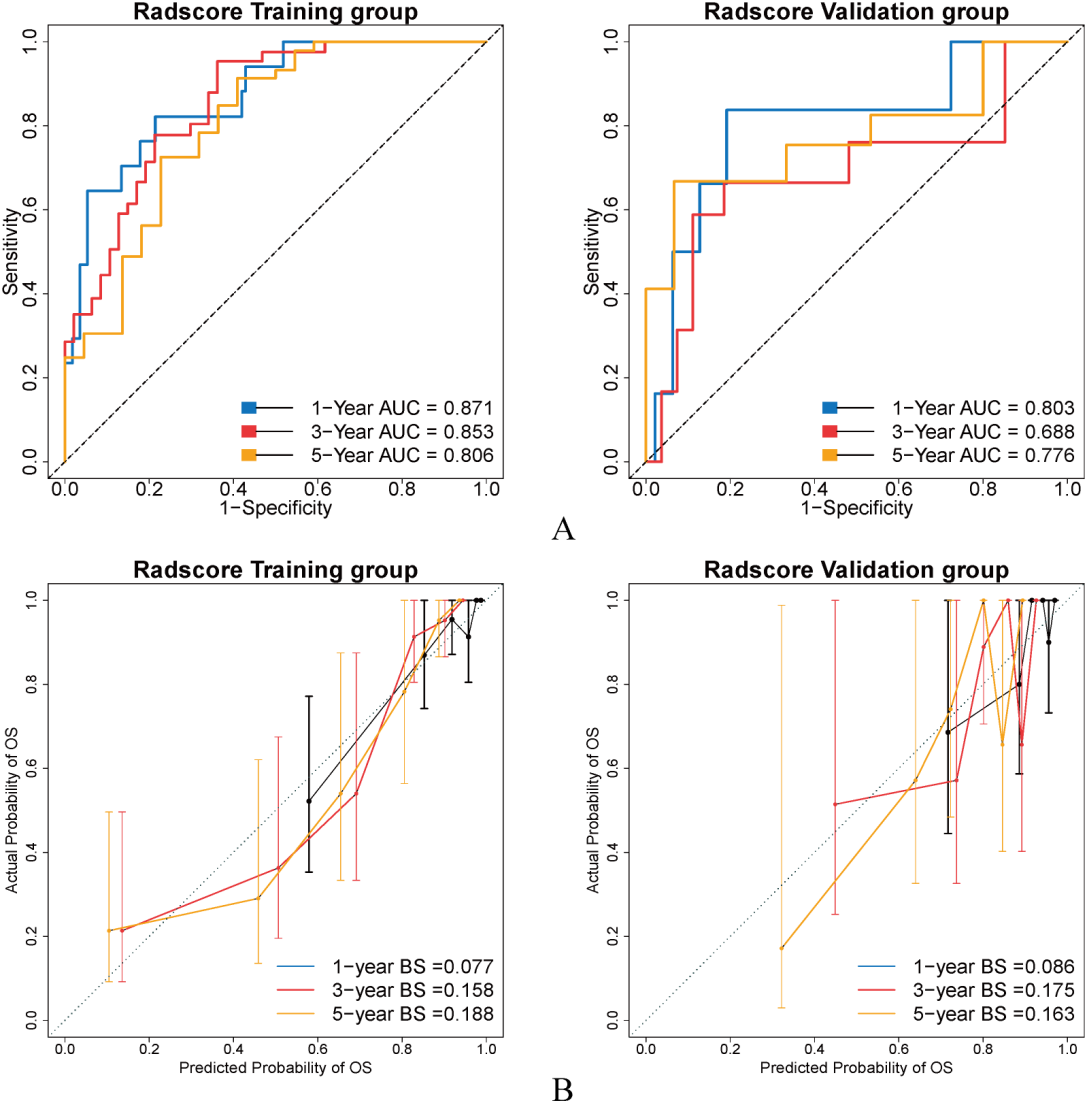
**(A)** ROC curve for 1, 3, 5-year OS prediction of GC patients with Radscore. **(B)** Calibration curve for 1, 3, 5-year OS prediction of GC patients with Radscore.

The results of univariate Cox regression (HR = 5.60, 95% CI: 3.60-8.71, p<0.001, **Table 1**) and multivariate Cox regression analysis (HR = 2.34, 95% CI: 1.3~4.21, p=0.005, **Table 2**) showed that Radscore was an independent predictor of OS in GC patients. Then, we further analyzed the efficacy of Radsocre in predicting OS of GC patients. In training group, the C-index of Radscore for predicting OS in GC patients is 0.839, while it is 0.729 in the validation group (**Figure 5A**). **Figure 6A** shows the ROC curves of Radscore for predicting 1, 3, and 5-year OS of GC patients, with AUC values of 0.871, 0.853, and 0.806 in the training group and 0.803, 0.688, and 0.776 in the validation group, respectively. **Figure 6B** shows the calibration curve of Radscore for predicting OS in GC patients. In the training group, the BS values for Radscore predicting 1, 3, and 5-year OS of GC patients were 0.077, 0.158, and 0.188, respectively, while in the validation group, the BS values were 0.086, 0.175, and 0.163, respectively.Whether in the training or the validation group, it showed a high consistency between the predicted 1, 3, 5 year OS of GC patients using the Radscore and the actual results. Overall, Radscore has good accuracy and goodness of fit in predicting the survival outcomes of GC patients.

### Screening of risk factors affecting the prognosis of GC and construction of the prognostic model

Based on the clinical data, pathological data, and Radscore of GC patients, univariate Cox regression analysis was used to screen risk factors associated with GC prognosis (**Table 1**). After using the VIF method to eliminate collinear variables (**Supplementary Table 3**), a total of 16 factors were screened (**Supplementary Table 4**) and included in further multivariate Cox regression analysis (Backward method). When the AIC (Akaike information criterion) value was minimized, a predictive model for GC survival outcomes was constructed. Finally, a simple clinical model and a combined model integrating clinical data, picrosirius red risk score, and Radscore were constructed. In the clinical model, the independent risk factors for predicting OS in GC patients include BMI, T stage, N stage, preoperative CA125, and preoperative CA72-4. Among them, N stage and preoperative CA72–4 did not have significant statistical significance, but were still used as independent variables in the model (**Table 4**). In the combined model, the independent risk factors for predicting OS in GC patients were BMI, picrosirius red risk score, T stage, preoperative CA125, and Radscore (**Table 2**). As the results showed, whether in the clinical model or combined model, BMI (HR = 0.85, 95% CI: 0.75~0.97, p=0.012), T stage (HR = 2.69, 95% CI: 1.46~4.92, p=0.001), preoperative CA125 (HR = 3.48, 95% CI: 1.14~10.62, p=0.028), Radscore (HR = 2.34, 95% CI: 1.3~4.21, p=0.005), and picrosirius red risk score (HR = 1.1, 95% CI: 1.02~1.18, p=0.013) are significant independent risk factors for predicting OS in GC patients.

**Table 4:** Multivariate Cox regression analysis and clinical model for predicting the prognosis of GC patients.

Factor	β	HR	Lower.95	Upper.95	P
BMI	-0.2	0.82	0.72	0.92	0.001
T stage	1.38	3.96	2.04	7.69	<0.001
N stage	0.31	1.36	1	1.85	0.053
preoperative CA125	1.67	5.3	1.99	14.12	0.001
preoperative CA72-4	0.57	1.76	0.9	3.48	0.101

### Evaluation of prognostic model and the construction of nomogram for GC

**Figure 5A** shows the C-index of each model in the training and validation groups. In the training and validation groups, the C-index of the combined model was 0.900 and 0.840, respectively, both of which were higher than the clinical model, picrosirius red risk score, and the Radscore. **Figure 5B** shows the ROC curves of each model for predicting 1, 3, 5-year OS of GC patients in the training and validation groups. The AUC of the combined model in the training group was 0.929, 0.911, and 0.88, respectively, while in the validation group was 0.802, 0.866, and 0.956, respectively. **Figure 5C** shows the calibration curve of each model for predicting OS in GC patients. The results showed that whether in the training or validation groups, there was a high consistency between the predicted 1, 3, 5 year OS of GC patients using the combined model and the actual results. The fluctuation amplitude of the calibration curve of the combined model is smaller than that of the clinical model, picrosirius staining score, and Radscore. In the training group, the BS values for combined model predicting 1, 3, and 5-year OS of GC patients were 0.066, 0.122, and 0.14, respectively, while in the validation group, the BS values were 0.078, 0.131, and 0.07, respectively. **Figure 5D** shows the decision curve of each model for predicting 1, 3, 5 year OS of GC patients. As the results shown, when the threshold probability is greater than 5%, the combined model can predict the 1-year OS well. When the threshold probability is 10% to 90%, it can predict the 3-year OS well. When the threshold probability is greater than 10%, it can predict the 5-year OS well. The combined model shows a greater net benefit than the clinical model, picrosirius red risk score, and Radscore in predicting 1, 3, and 5-year OS in GC patients. Overall, the combined model shows higher predictive efficacy for the prognosis of GC patients than the clinical model, picrosirius red risk score or Radscore.

**Figure 7:**
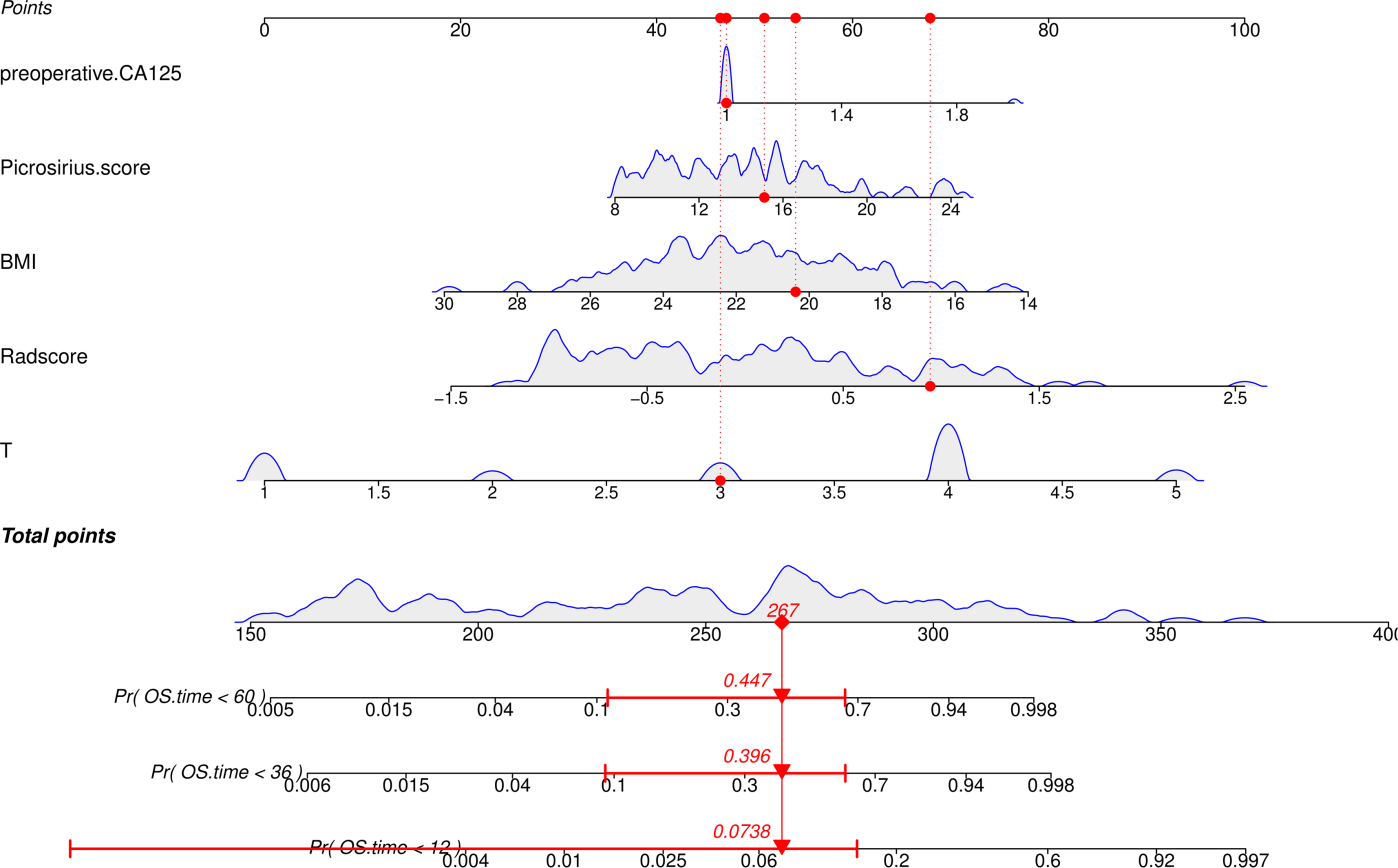
Nomogram for predicting OS in GC patients using the combined model.

For clinical application, this study constructed a nomogram (**Figure 7**) based on the combined model. In the nomogram, Points correspond to the score of each variable. The Total points is the sum of the Points. The Total points of each GC patient corresponds to his/her predicted risk of death. As shown in **Figure 7**, when the Total points is 267, the 1, 3, and 5-year mortality rate of GC patients are 0.0738, 0.396, and 0.447, respectively.

## Discussion

Due to the heterogeneity of GC, there is still no good method to accurately predict the prognosis of GC patients. Collagen represents the most abundant protein within the tumor microenvironment (TME), exerting a significant impact on TME structure, tumor cell metabolism, proliferation and malignant progression (33). It may therefore be posited that an understanding of the role of collagen in gastric carcinogenesis could provide a novel framework for the development of therapeutic strategies and the improvement of outcomes in GC. Furthermore, it assists in the identification of GC patients who are at an elevated risk of recurrence and a poorer prognosis, thereby enabling clinicians to enhance follow-up strategies.

Our study is in accordance with previous findings that have demonstrated a correlation between collagen remodelling and the development and progression of GC (9, 10). According to collagen genes expression, GC patients were classified into two subtypes. Consistent with previous findings (11), high expression of collagen genes was associated with poor prognosis regardless of the stage of GC. Further, in order to allow rapid identification of collagen subtypes in GC for clinical applications, this study used picrosirius red staining to specifically stain the collagen components of GC tissues and constructed a picrosirius red risk score. The results of the univariate and multivariate Cox regression analyses demonstrated that the picrosirius red risk score was an independent risk factor affecting the prognosis of GC. The efficacy and clinical utility of predicting OS in GC patients by the picrosirius red risk score were demonstrated by ROC and calibration curves. The mean value of the picrosirius red risk score was employed to identify high and low picrosirius red staining subgroups of GC, and the differences in survival outcomes between the subgroups were demonstrated. The role of collagen in predicting the prognosis of GC patients was further validated at the protein level, as well as the results of bioinformatics analysis at the gene level. Furthermore, several studies have indicated that collagen expression levels are associated with the prognosis of GC. Zhou et al. used second harmonic generation imaging technology to detect collagen fibers of GC and showed that collagen width (AUC = 0.741 [95% CI: 0.675-0.807], p<0.001), collagen density (AUC = 0.632 [95% CI: 0.559-0.706], p=0.001) and collagen length (AUC = 0.672, [95% CI: 0.602-0.742], p<0.001) were all effective predictors of 5-year OS in GC (32). Chen et al. used multiphoton imaging to extract collagen features of GC and construct collagen signature, and demonstrated that high collagen signature were associated with poor prognosis of GC (training group: HR = 3.54 [95% CI: 2.41-5.20], p<0.001), validation group: HR = 3.24 [95% CI: 2.33-4.50], p<0.001) (31), also associated with peritoneal metastasis of GC (univariate Cox regression: HR = 4.11 [95% CI: 2.58-6.56], p<0.001; multivariate Cox regression: HR = 2.49 [95% CI: 1.52-4.08], p<0.001) (34). Furthermore, the findings of this study indicate that picrosirius red staining may serve as a rapid and effective method for assessing the extent of collagen enrichment in GC. It can be combined with existing pathological analyses to identify patients with high-risk GC.

With the development of radiology, CT scans are a primary tool for assessing tumors (35). However, conventional techniques are only capable of identifying the fundamental structural attributes of the tumor at the outset, yet they are unable to ascertain the underlying quantitative characteristics of the tumor. Radiomics enables the high-throughput extraction of a range of quantitative features from medical images, facilitating subsequent analysis (36). It is able to construct models through the utilisation of machine learning or artificial intelligence algorithms, thereby facilitating the process of clinical decision-making (16, 37–41). In this study, we employed preoperative enhanced CT arterial phase images of GC patients to perform radiomic analysis. Following univariate Cox regression and LASSO regression analysis, a total of 11 radiomic features were identified and used to construct Radscore. Among them, the log.sigma.3.mm.3D_glszm_SizeZoneNonUniformity feature has the largest coefficient, which measures the variability of the size zone volumes throughout the image and represents the tumor heterogeneity. Higher value represents greater tumor heterogeneity, reflecting the fact that tumors may have lower adhesion, higher invasiveness and greater self-repairing ability (42). Through univariate and multivariate Cox regression analyses, we demonstrated that the Radscore was an independent risk factor affecting the prognosis of GC. The efficacy and clinical utility of predicting OS in GC patients by the radiomic score were demonstrated by ROC and calibration curves, which is consistent with previous studies (41, 43). Therefore, information from high-throughput radiomic features can accurately assess both the heterogeneity of GC and the survival of GC patients, which can help clinicians identify patients with poor prognosis more quickly.

In clinical practice, an increasing amount of medical information about the patient is accessible to the physician, including clinical, pathological and imaging data. No single piece of data can fully characterise cancers and make an accurate judgement of patients’ outcome (41, 44, 45). Therefore, the prognostic model constructed by combining multiple data from patients is more capable of comprehensively evaluating the heterogeneity of tumors and patients, thus better predicting the survival outcomes of GC patients.

Through univariate Cox regression and multivariate Cox regression analysis, this study constructed a clinical model and a combined model for predicting GC OS. Regardless of the model, BMI, T stage, preoperative CA125, picrosirius red risk score and Radsore were independent risk factors for predicting OS in patients with GC. Low BMI were risk factors affecting the prognosis of GC, which is consistent with the results of previous studies (46, 47). The difference in nutritional reserves may be the reason for the different prognoses of GC caused by BMI and weight (48). In terms of pathological indicators, more advanced T stage, N stage and higher picrosirius red risk score were associated with poor prognosis in GC. This is consistent with previous studies and the above results. T stage represents the depth of tumor infiltration, and higher T stages all represent larger tumor size and higher degree of infiltration and poorer prognosis for patients. Furthermore, several studies have demonstrated a positive correlation between serum tumor marker levels and GC prognosis (49–51). CA125 is a macromolecular polysaccharide protein, and several studies have shown that the expression level of serum CA125 correlates with peritoneal metastasis (52), as well as with poor prognosis in GC (53). Among all factors, Radsore had the highest HR, further demonstrating the accuracy of high-throughput radiomic features for identifying GCs with poor prognosis.

The C-index, ROC curve, calibration curve, and decision curve analysis all demonstrated that the combined model for predicting OS in patients with GC exhibited superior predictive efficacy, goodness of fit, and clinical benefit compared to clinical data, picrosirius red risk score, or Radsore alone. The results indicate the clinical necessity of combining clinical, pathological, and imaging datasets to comprehensively assess the patient’s condition. This is consistent with the findings of multiple previous studies. Jia et al. demonstrated that the combined model (training set AUC = 0.851, validation set AUC = 0.813) constructed by combining the radiomic and clinical data was more effective in predicting OS in GC patients than the radiomic data (training set AUC = 0.829, validation set AUC = 0.779) or clinical data (training set AUC = 0.820, validation set AUC = 0.669) alone (54). The study also presents a nomogram that clinicians can easily employ to enhance their ability to anticipate the prognosis of GC patients. This will facilitate more informed clinical decision-making and follow-up strategies, ultimately improving patient outcomes and prolonging survival.

This study has several limitations that should be acknowledged. First, the sample size was relatively small and all patients were derived from a single center, which may introduce potential bias and limit the generalizability of the model. In addition, the delineation of picrosirius red staining regions in tumor sections and the segmentation of ROIs in CT images were performed manually, which may introduce inter-observer variability despite efforts to ensure consistency between physicians. Furthermore, the radiomics analysis in this study was mainly based on arterial phase images, while radiomic features from portal venous and venous phases were not comprehensively explored, which may have limited the completeness of feature extraction. Finally, the validation cohort used in this study was an internal validation set rather than an external multicenter cohort. Differences in CT scanning protocols across institutions may affect imaging characteristics and potentially influence radiomic feature extraction. Therefore, larger multicenter studies with standardized imaging protocols and external validation are warranted to further confirm the robustness and clinical applicability of our findings.

## Conclusion

High collagen density influences the occurrence and development of GC. The prognosis of GC patients with different collagen densities varies, with those in the Collagen-High group exhibiting a less favorable prognosis. Picrosirius red staining reflects the enrichment of collagen components in the ECM. The picrosirius red risk score serves as an independent risk factor for predicting the prognosis of GC patients. A significant disparity was observed in the survival rate of GC patients between those with high and low picrosirius red risk scores. The Radscore reflects the high-throughput characteristics of CT images of GC and is an independent risk factor for predicting the prognosis of GC patients. The prognostic model constructed by combining the clinical data, the picrosirius red risk score, and the Radscore of GC patients demonstrated good predictive efficacy, goodness-of-fit, and clinical benefit.

## Data Availability

The datasets presented in this study can be found in online repositories. The names of the repository/repositories and accession number(s) can be found in the article/**Supplementary Material**.
