# Supplementary material for: A novel combined model integrating collagen properties, radiomics and clinical data to predict gastric cancer prognosis
Source: Front Oncol. 2026 Apr 10;16:1801350. doi: 10.3389/fonc.2026.1801350 (PMC13105883; doi:10.3389/fonc.2026.1801350)
Supplement: Supplementary file 1 [file DataSheet1.docx]

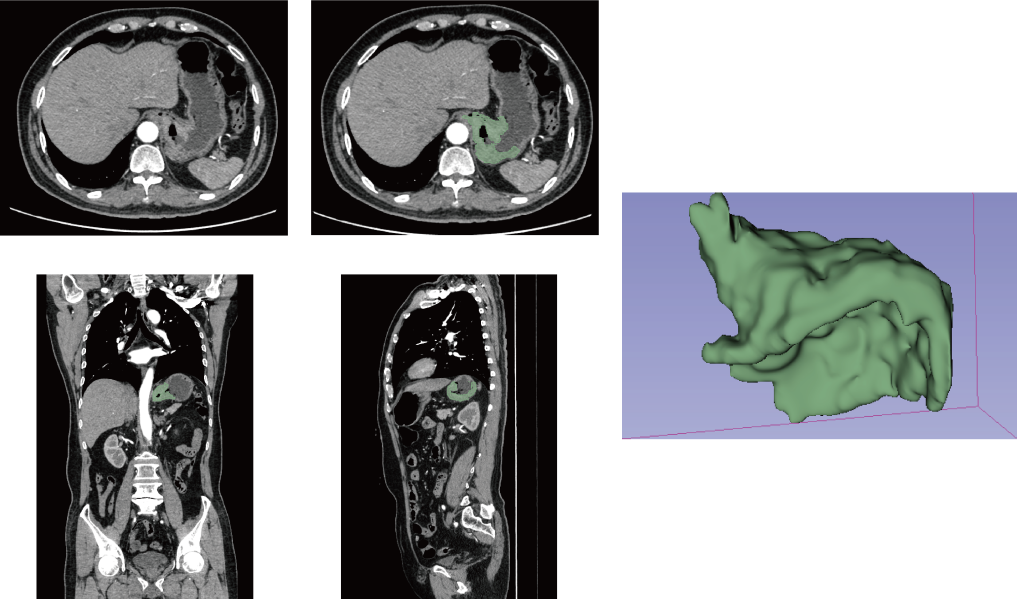


Figure S1: The ROI of the enhanced CT arterial phase images of the enrolled GC patients.


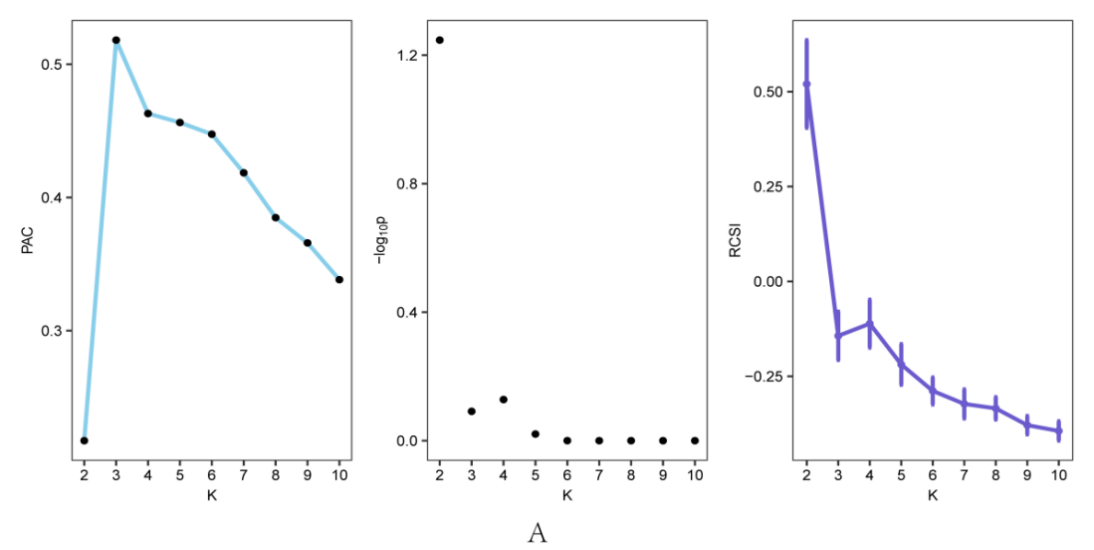


Figure S2: A Proportional of Ambiguous Clustering Score(left), p-values (medium), and Relative Cluster Stability Index (right) for unsupervised clustering of TCGA-STAD samples.


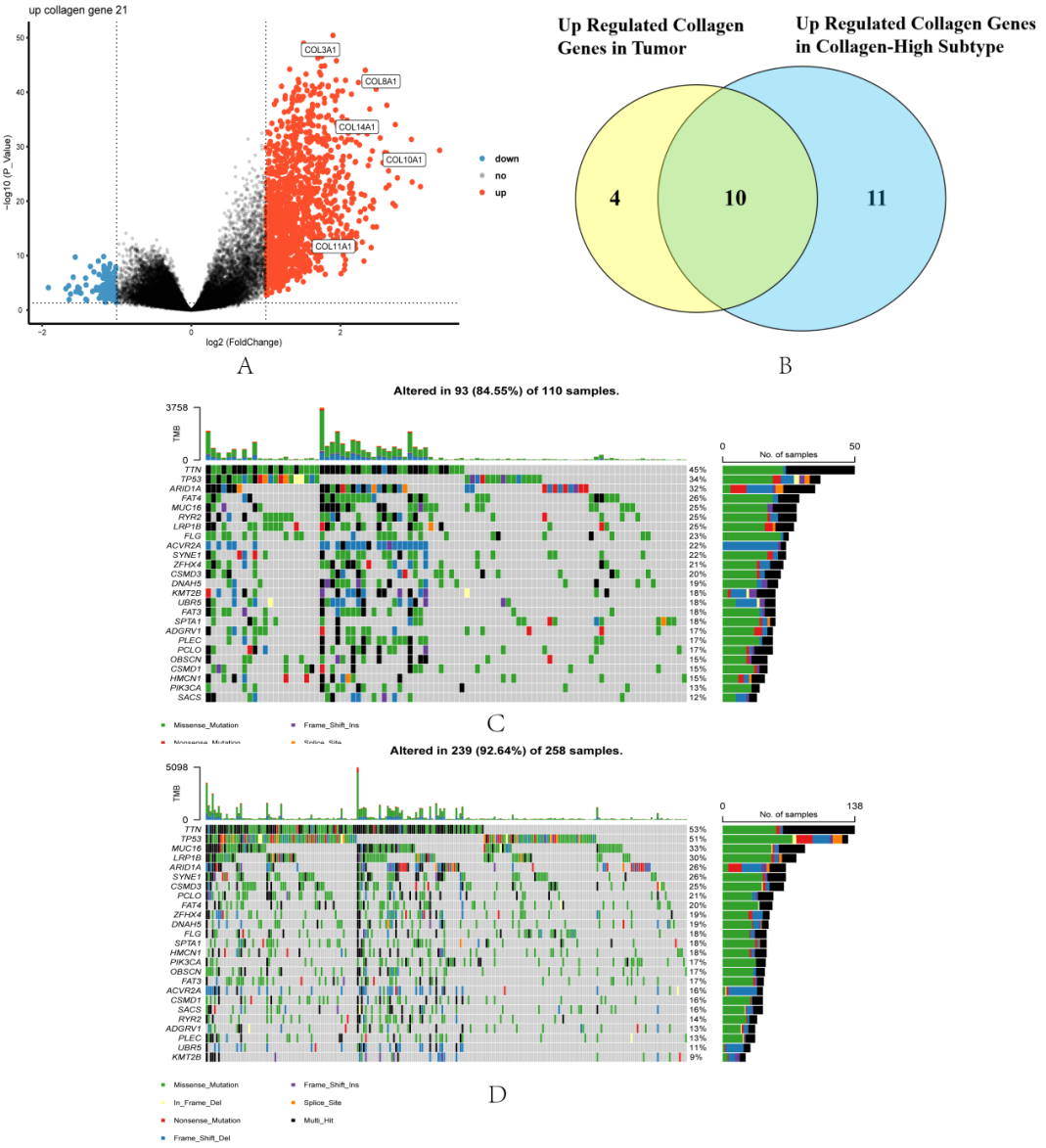


Figure S3: A Volcano plot displaying the outcomes of the differential analysis carried out on collagen subtypes for the TCGA cohort, with the top 5 up-regulated collagen genes specially highlighted. B Venn plot showing the intersecting genes between the up-regulated collagen genes in GC and in Collagen-High group (Upregulated collagen genes identified in tumor tissues included COL21A1, COL25A1, COL24A1, COL10A1, COL11A1, COL12A1, COL14A1, COL15A1, COL16A1, COL1A1, COL1A2, COL3A1, COL4A2, COL4A4, COL5A1, COL5A2, COL6A1, COL6A2, COL6A3, COL8A1, and COL8A2. Upregulated collagen genes identified in the Collagen-High subtype included COL10A1, COL11A1, COL12A1, COL1A1, COL1A2, COL3A1, COL4A1, COL5A1, COL5A2, COL6A3, COL7A1, COL8A1, COL9A1, and COL22A1).
