# Supplementary material for: A novel combined model integrating collagen properties, radiomics and clinical data to predict gastric cancer prognosis
Source: Front Oncol. 2026 Apr 10;16:1801350. doi: 10.3389/fonc.2026.1801350 (PMC13105883; doi:10.3389/fonc.2026.1801350)
Supplement: Supplementary file 3 [file Table1.docx]

Supplement Table 1 Comparison of general information between high and low picrosirius red staining groups

|  | low picrosirius red staining group | high picrosirius red staining group | p |
| --- | --- | --- | --- |
| N | 104 | 92 |  |
| Sex，n（%） |  |  |  |
| female | 39 ( 37.5) | 40 ( 43.5) | 0.48 |
| male | 65 ( 62.5) | 52 ( 56.5) |  |
| Age，median[IQR] | 57.00 [48.75, 65.00] | 56.50 [44.00, 63.25] | 0.444 |
| Height (cm)，median[IQR] | 165.00 [160.00, 170.00] | 165.00 [157.00, 170.00] | 0.606 |
| Weight (kg)，mean (SD) | 59.92 (10.91) | 58.58 (10.87) | 0.391 |
| BMI（kg/m^2）,mean (SD) | 22.07 (2.96) | 21.82 (3.02) | 0.563 |
| History of smoking, n（%） |  |  |  |
| No | 71 ( 68.3) | 58 ( 63.0) | 0.536 |
| Yes | 33 ( 31.7) | 34 ( 37.0) |  |
| Tumor location，n（%） |  |  |  |
| Antrum | 58 ( 55.8) | 48 ( 52.2) | 0.801 |
| Body | 26 ( 25.0) | 27 ( 29.3) |  |
| Cardia | 5 ( 4.8) | 7 ( 7.6) |  |
| Fundus | 7 ( 6.7) | 4 ( 4.3) |  |
| Gastroesophageal junction | 8 ( 7.7) | 6 ( 6.5) |  |
| Surgery method, n（%） |  |  |  |
| Laparoscope | 84 ( 80.8) | 66 ( 71.7) | 0.223 |
| Conversion to open laparotomy | 2 ( 1.9) | 4 ( 4.3) |  |
| Open surgery | 3 ( 2.9) | 8 ( 8.7) |  |
| Robot-assisted | 15 ( 14.4) | 14 ( 15.2) |  |
| Operation time (min)，median[IQR] | 272.50 [235.00, 301.25] | 265.00 [225.00, 306.25] | 0.75 |
| Blood loss (ml), median[IQR] | 100.00 [50.00, 150.00] | 100.00 [50.00, 200.00] | 0.446 |
| Number of lymph nodes, median [IQR] | 2.00 [0.00, 9.00] | 7.50 [1.75, 15.00] | 0.001 |
| Picrosirius red risk score, median[IQR] | 11.00 [9.86, 12.50] | 16.94 [15.58, 19.13] | <0.001 |
| TNM stage, n（%） |  |  |  |
| I | 34 ( 32.7) | 19 ( 20.7) | 0.038 |
| II | 18 ( 17.3) | 9 ( 9.8) |  |
| III | 48 ( 46.2) | 56 ( 60.9) |  |
| IV | 4 ( 3.8) | 8 ( 8.7) |  |
| T stage, n（%） |  |  |  |
| 1 | 28 ( 26.9) | 17 ( 18.5) | 0.045 |
| 2 | 12 ( 11.5) | 9 ( 9.8) |  |
| 3 | 12 ( 11.5) | 13 ( 14.1) |  |
| 4a | 49 ( 47.1) | 40 ( 43.5) |  |
| 4b | 3 ( 2.9) | 13 ( 14.1) |  |
| N stage, n（%） |  |  |  |
| 0 | 40 ( 38.5) | 17 ( 18.5) | 0.002 |
| 1 | 16 ( 15.4) | 10 ( 10.9) |  |
| 2 | 14 ( 13.5) | 12 ( 13.0) |  |
| 3a | 14 ( 13.5) | 32 ( 34.8) |  |
| 3b | 20 ( 19.2) | 21 ( 22.8) |  |
| M stage, n（%） |  |  |  |
| 0 | 99 ( 96.1) | 84 ( 91.3) | 0.233 |
| 1 | 4 ( 3.9) | 8 ( 8.7) |  |
| Her.2, n（%） |  |  |  |
| 0 | 43 ( 41.3) | 39 ( 42.4) | 0.694 |
| 1+ | 33 ( 31.7) | 31 ( 33.7) |  |
| 2+ | 25 ( 24.0) | 17 ( 18.5) |  |
| 3+ | 3 ( 2.9) | 5 ( 5.4) |  |
| Pathology, n（%） |  |  |  |
| Intermediate to highly differentiated adenocarcinoma | 34 ( 32.7) | 30 ( 32.6) | 1 |
| poorly differentiated adenocarcinoma | 70 ( 67.3) | 62 ( 67.4) |  |
| Postoperative hospital days, median[IQR] | 10.00 [8.00, 13.00] | 10.50 [8.00, 14.00] | 0.587 |
| Chemotherapy, n（%） |  |  |  |
| No | 34 ( 32.7) | 18 ( 19.6) | 0.055 |
| Yes | 70 ( 67.3) | 74 ( 80.4) |  |
| Follow-up time (months), median [IQR] | 31.00 [19.00, 57.50] | 23.50 [13.00, 46.00] | 0.008 |
| Death，n（%） |  |  |  |
| No | 86 ( 82.7) | 54 ( 58.7) | <0.001 |
| Yes | 18 ( 17.3) | 38 ( 41.3) |  |
| Preoperative leukocyte(X10^9/L)，median[IQR] | 5.78 [5.01, 6.91] | 6.10 [4.91, 7.39] | 0.463 |
| Preoperative neutrophil (X10^9/L), median[IQR] | 3.30 [2.58, 4.51] | 3.39 [2.78, 4.63] | 0.414 |
| Preoperative lymphocyte(X10^9/L), median [IQR] | 1.70 [1.31, 2.12] | 1.77 [1.33, 2.13] | 0.586 |
| Preoperative hemoglobin (g/L)，median[IQR] | 124.00 [99.00, 136.00] | 124.00 [103.50, 138.00] | 0.698 |
| Preoperative platelet (X10^9/L), median [IQR] | 260.50 [225.75, 313.75] | 260.00 [214.75, 326.50] | 0.915 |
| Preoperative neutrophil to lymphocyte ratio, median[IQR] | 2.00 [1.43, 2.73] | 2.02 [1.59, 2.83] | 0.694 |
| Preoperative platelet to lymphocyte ratio, median[IQR] | 167.11 [114.72, 205.39] | 147.15 [109.24, 222.11] | 0.584 |
| Preoperative albumin(g/L), mean(SD) | 37.91 (4.63) | 38.98 (4.16) | 0.091 |
| Preoperative globulin(g/L), mean(SD) | 29.04 (4.93) | 29.06 (4.82) | 0.984 |
| Preoperative total bilirubin (μmol/L)，median[IQR] | 10.40 [8.55, 12.93] | 10.35 [7.70, 14.83] | 0.718 |
| Preoperative ALT(U/L)，median[IQR] | 14.00 [11.00, 20.25] | 13.00 [10.00, 17.00] | 0.155 |
| Preoperative AST(U/L)，median[IQR] | 19.00 [15.00, 23.00] | 18.00 [15.00, 21.00] | 0.469 |
| Preoperative urea(mmol/L)，median[IQR] | 4.90 [4.10, 5.93] | 4.90 [3.98, 6.00] | 0.877 |
| Preoperative creatinine (μmol/L)，median[IQR] | 82.00 [70.75, 90.00] | 78.50 [68.75, 90.25] | 0.556 |
| Preoperative glucose(mmol/L)，median[IQR] | 4.80 [4.40, 5.30] | 5.10 [4.57, 5.53] | 0.043 |
| Preoperative AFP(μg/L)，median[IQR] | 2.40 [1.90, 3.32] | 2.32 [1.72, 3.19] | 0.393 |
| Preoperative CEA(μg/L)，median[IQR] | 1.95 [1.20, 3.50] | 2.10 [1.30, 3.92] | 0.317 |
| Preoperative CA125(U/mL)，median[IQR] | 10.20 [6.95, 14.22] | 10.60 [8.17, 16.75] | 0.112 |
| Preoperative CA19-9(U/mL)，median[IQR] | 10.40 [6.27, 18.33] | 10.80 [6.50, 22.57] | 0.485 |
| Preoperative CA72-4(U/mL)， median[IQR] | 1.40 [1.00, 3.05] | 2.00 [1.23, 4.80] | 0.021 |
| Postoperative leukocyte (X10^9/L)，median[IQR] | 7.82 [6.60, 9.51] | 7.97 [6.23, 10.96] | 0.575 |
| Postoperative neutrophil (X10^9/L)，median[IQR] | 5.70 [4.54, 7.38] | 5.72 [4.12, 8.53] | 0.493 |
| Postoperative lymphocyte (X10^9/L)，median[IQR] | 1.16 [0.91, 1.38] | 1.15 [0.86, 1.50] | 0.659 |
| Postoperative hemoglobin (g/L)，median[IQR] | 102.00 [88.50, 117.00] | 102.00 [87.00, 115.00] | 0.766 |
| Postoperative platelet (X10^9/L)，median[IQR] | 273.50 [202.75, 346.25] | 294.50 [216.75, 385.00] | 0.185 |
| Postoperative neutrophil to lymphocyte ratio，median[IQR] | 5.03 [3.81, 6.58] | 5.25 [3.23, 7.22] | 0.735 |
| Postoperative platelet to lymphocyte ratio，median[IQR] | 246.48 [174.15, 318.24] | 224.25 [174.26, 325.91] | 0.93 |
| Postoperative albumin (g/L)，mean(SD) | 33.99 (4.53) | 34.05 (4.38) | 0.924 |
| Postoperative globulin(g/L)，median[IQR] | 26.30 [23.17, 29.65] | 26.65 [24.20, 29.75] | 0.585 |
| Postoperative total bilirubin (μmol/L)，median[IQR] | 11.10 [6.90, 16.22] | 11.95 [8.52, 14.82] | 0.64 |
| Postoperative ALT(U/L)，median[IQR] | 29.00 [20.00, 44.00] | 30.00 [21.75, 42.00] | 0.909 |
| Postoperative AST，median[IQR] | 24.00 [17.75, 35.00] | 24.00 [18.75, 30.25] | 0.531 |
| Postoperative urea (mmol/L)，median[IQR] | 5.85 [4.97, 7.03] | 5.65 [4.60, 7.20] | 0.286 |
| Postoperative creatinine (μmol/L)，median[IQR] | 71.00 [62.00, 82.00] | 70.00 [61.00, 83.00] | 0.775 |
| Postoperative glucose (mmol/L)，median[IQR] | 5.50 [4.88, 6.40] | 5.30 [4.77, 6.25] | 0.52 |

Supplement Table 2 Comparison of general information between training and validation groups

|  | Training group | Validation group | p |
| --- | --- | --- | --- |
| N | 138 | 58 |  |
| Sex，n（%） |  |  |  |
| female | 56 ( 40.6) | 23 ( 39.7) | 1 |
| male | 82 ( 59.4) | 35 ( 60.3) |  |
| Age，median[IQR] | 57.00 [46.00, 64.00] | 57.50 [49.25, 63.75] | 0.744 |
| Height (cm)，median[IQR] | 165.00 [158.00, 170.00] | 162.50 [160.00, 168.00] | 0.231 |
| Weight (kg)，mean (SD) | 58.92 (10.25) | 60.16 (12.30) | 0.468 |
| BMI（kg/m^2）,mean (SD) | 21.76 (2.79) | 22.44 (3.38) | 0.146 |
| History of smoking, n（%） |  |  |  |
| No | 87 ( 63.0) | 42 ( 72.4) | 0.272 |
| Yes | 51 ( 37.0) | 16 ( 27.6) |  |
| Tumor location，n（%） |  |  |  |
| Antrum | 74 ( 53.6) | 32 ( 55.2) | 0.551 |
| Body | 35 ( 25.4) | 18 ( 31.0) |  |
| Cardia | 10 ( 7.2) | 2 ( 3.4) |  |
| Fundus | 7 ( 5.1) | 4 ( 6.9) |  |
| Gastroesophageal junction | 12 ( 8.7) | 2 ( 3.4) |  |
| Surgery method, n（%） |  |  |  |
| Laparoscope | 106 ( 76.8) | 44 ( 75.9) | 0.985 |
| Conversion to open laparotomy | 4 ( 2.9) | 2 ( 3.4) |  |
| Open surgery | 8 ( 5.8) | 3 ( 5.2) |  |
| Robot-assisted | 20 ( 14.5) | 9 ( 15.5) |  |
| Operation time (min)，median[IQR] | 270.00 [226.25, 300.00] | 267.50 [231.25, 323.00] | 0.543 |
| Blood loss (ml), median[IQR] | 100.00 [50.00, 150.00] | 100.00 [50.00, 150.00] | 0.477 |
| Number of lymph nodes, median [IQR] | 4.50 [0.00, 15.00] | 3.00 [0.00, 10.50] | 0.238 |
| Picrosirius red risk score, median[IQR] | 13.85 [10.83, 16.69] | 13.22 [10.80, 16.85] | 0.825 |
| TNM stage, n（%） |  |  |  |
| I | 33 ( 23.9) | 20 ( 34.5) | 0.323 |
| II | 22 ( 15.9) | 5 ( 8.6) |  |
| III | 75 ( 54.3) | 29 ( 50.0) |  |
| IV | 8 ( 5.8) | 4 ( 6.9) |  |
| T stage, n（%） |  |  |  |
| 1 | 31 ( 22.5) | 14 ( 24.1) | 0.348 |
| 2 | 11 ( 8.0) | 10 ( 17.2) |  |
| 3 | 20 ( 14.5) | 5 ( 8.6) |  |
| 4a | 64 ( 46.4) | 25 ( 43.1) |  |
| 4b | 12 ( 8.7) | 4 ( 6.9) |  |
| N stage, n（%） |  |  |  |
| 0 | 36 ( 26.1) | 21 ( 36.2) | 0.488 |
| 1 | 21 ( 15.2) | 5 ( 8.6) |  |
| 2 | 19 ( 13.8) | 7 ( 12.1) |  |
| 3a | 31 ( 22.5) | 15 ( 25.9) |  |
| 3b | 31 ( 22.5) | 10 ( 17.2) |  |
| M stage, n（%） |  |  |  |
| 0 | 129 ( 94.2) | 54 ( 93.1) | 0.753 |
| 1 | 8 ( 5.8) | 4 ( 6.9) |  |
| Her.2, n（%） |  |  |  |
| 0 | 54 ( 39.1) | 28 ( 48.3) | 0.284 |
| 1+ | 49 ( 35.5) | 15 ( 25.9) |  |
| 2+ | 31 ( 22.5) | 11 ( 19.0) |  |
| 3+ | 4 ( 2.9) | 4 ( 6.9) |  |
| Pathology, n（%） |  |  |  |
| Intermediate to highly differentiated adenocarcinoma | 46 ( 33.3) | 18 ( 31.0) | 0.884 |
| poorly differentiated adenocarcinoma | 92 ( 66.7) | 40 ( 69.0) |  |
| Postoperative hospital days, median[IQR] | 10.00 [8.00, 13.00] | 11.00 [8.00, 13.75] | 0.737 |
| Chemotherapy, n（%） |  |  |  |
| No | 34 ( 24.6) | 18 ( 31.0) | 0.454 |
| Yes | 104 ( 75.4) | 40 ( 69.0) |  |
| Follow-up time (months), median [IQR] | 26.00 [14.25, 46.00] | 27.00 [16.50, 59.25] | 0.275 |
| Death，n（%） |  |  |  |
| No | 34 ( 24.6) | 19 ( 32.8) | 0.321 |
| Yes | 104 ( 75.4) | 39 ( 67.2) |  |
| Preoperative leukocyte(X10^9/L)，median[IQR] | 6.04 [5.00, 7.14] | 5.86 [4.70, 7.73] | 0.677 |
| Preoperative neutrophil (X10^9/L), median[IQR] | 3.38 [2.70, 4.60] | 3.20 [2.56, 4.62] | 0.575 |
| Preoperative lymphocyte(X10^9/L), median [IQR] | 1.70 [1.31, 2.12] | 1.74 [1.26, 2.17] | 0.683 |
| Preoperative hemoglobin (g/L)，median[IQR] | 125.00 [103.25, 136.00] | 123.50 [98.50, 141.25] | 0.96 |
| Preoperative platelet (X10^9/L), median [IQR] | 260.50 [225.75, 322.00] | 252.50 [215.50, 302.00] | 0.231 |
| Preoperative neutrophil to lymphocyte ratio, median[IQR] | 2.09 [1.46, 2.72] | 1.86 [1.59, 2.86] | 0.938 |
| Preoperative platelet to lymphocyte ratio, median[IQR] | 161.23 [112.99, 214.31] | 141.97 [114.21, 213.57] | 0.437 |
| Preoperative albumin(g/L), mean(SD) | 38.32 (4.48) | 38.62 (4.36) | 0.665 |
| Preoperative globulin(g/L), mean(SD) | 29.16 (5.13) | 28.79 (4.21) | 0.625 |
| Preoperative total bilirubin (μmol/L)，median[IQR] | 10.35 [8.00, 13.97] | 11.05 [8.90, 14.68] | 0.31 |
| Preoperative ALT(U/L)，median[IQR] | 13.50 [11.00, 19.00] | 12.00 [9.00, 18.50] | 0.226 |
| Preoperative AST(U/L)，median[IQR] | 18.00 [15.00, 22.00] | 18.00 [15.00, 20.75] | 0.564 |
| Preoperative urea(mmol/L)，median[IQR] | 4.95 [4.10, 6.00] | 4.70 [3.82, 5.97] | 0.345 |
| Preoperative creatinine (μmol/L)，median[IQR] | 79.50 [69.25, 91.75] | 82.00 [72.25, 88.00] | 0.431 |
| Preoperative glucose(mmol/L)，median[IQR] | 4.90 [4.40, 5.50] | 4.90 [4.60, 5.57] | 0.387 |
| Preoperative AFP(μg/L)，median[IQR] | 2.41 [1.78, 3.32] | 2.16 [1.77, 3.28] | 0.745 |
| Preoperative CEA(μg/L)，median[IQR] | 2.20 [1.30, 3.68] | 1.95 [1.30, 3.88] | 0.596 |
| Preoperative CA125(U/mL)，median[IQR] | 10.20 [7.62, 15.17] | 10.70 [7.15, 15.10] | 0.767 |
| Preoperative CA19-9(U/mL)，median[IQR] | 10.45 [5.67, 21.00] | 10.80 [6.98, 22.05] | 0.466 |
| Preoperative CA72-4(U/mL)， median[IQR] | 1.75 [1.00, 4.35] | 1.55 [1.00, 2.98] | 0.251 |
| Postoperative leukocyte (X10^9/L)，median[IQR] | 7.89 [6.31, 10.25] | 7.94 [6.72, 9.22] | 0.877 |
| Postoperative neutrophil (X10^9/L)，median[IQR] | 5.70 [4.12, 7.76] | 5.72 [4.58, 7.14] | 0.835 |
| Postoperative lymphocyte (X10^9/L)，median[IQR] | 1.14 [0.88, 1.44] | 1.22 [0.91, 1.43] | 0.488 |
| Postoperative hemoglobin (g/L)，median[IQR] | 101.00 [86.25, 113.00] | 105.00 [89.25, 117.75] | 0.247 |
| Postoperative platelet (X10^9/L)，median[IQR] | 286.50 [206.00, 379.25] | 285.00 [194.25, 339.75] | 0.204 |
| Postoperative neutrophil to lymphocyte ratio，median[IQR] | 4.97 [3.68, 6.98] | 5.38 [3.71, 6.61] | 0.747 |
| Postoperative platelet to lymphocyte ratio，median[IQR] | 248.31 [177.09, 334.52] | 212.88 [167.17, 292.74] | 0.113 |
| Postoperative albumin (g/L)，mean(SD) | 33.93 (4.54) | 34.22 (4.27) | 0.674 |
| Postoperative globulin(g/L)，median[IQR] | 26.45 [23.30, 29.48] | 26.55 [24.30, 30.90] | 0.334 |
| Postoperative total bilirubin (μmol/L)，median[IQR] | 11.35 [7.23, 15.57] | 12.05 [8.70, 17.25] | 0.503 |
| Postoperative ALT(U/L)，median[IQR] | 29.00 [20.00, 43.50] | 30.00 [22.00, 40.75] | 0.748 |
| Postoperative AST，median[IQR] | 23.00 [17.25, 32.00] | 24.00 [19.50, 37.50] | 0.403 |
| Postoperative urea (mmol/L)，median[IQR] | 5.70 [4.73, 7.18] | 5.80 [4.75, 7.05] | 0.925 |
| Postoperative creatinine (μmol/L)，median[IQR] | 70.00 [62.00, 83.00] | 72.50 [60.25, 82.75] | 0.845 |
| Postoperative glucose (mmol/L)，median[IQR] | 5.50 [4.80, 6.40] | 5.45 [4.82, 6.27] | 0.841 |

Supplementary Table 3: VIF analysis for significant factors in univariate Cox regression

| Factor | VIF |
| --- | --- |
| Weight | 3.630306982 |
| BMI | 3.501262708 |
| Number of lymph nodes | 3.891790284 |
| Picrosirius red risk score | 1.321585082 |
| TNM stage | 13.78495892 |
| T stage | 5.707265473 |
| N stage | 8.936539741 |
| M stage | 2.216104189 |
| Pathology | 1.27131618 |
| Chemotherapy | 2.622286775 |
| Preoperative lymphocyte | 1.328064226 |
| Preoperative AFP | 1.218824147 |
| Preoperative CA125 | 1.369318317 |
| Preoperative CA72-4 | 1.301060997 |
| Postoperative hemoglobin | 1.375107278 |
| Postoperative neutrophil to lymphocyte ratio | 1.162995957 |
| Postoperative platelet to lymphocyte ratio | 1.398310939 |
| Radscore | 2.829808573 |

Supplementary Table 4: VIF analysis for significant factors in univariate Cox regression after removing collinearity factors

| Factor | VIF |
| --- | --- |
| Weight | 3.557749065 |
| BMI | 3.398383243 |
| Picrosirius red risk score | 1.310145745 |
| T stage | 2.87291934 |
| N stage | 2.426305068 |
| M stage | 1.393323032 |
| Pathology | 1.255250891 |
| Chemotherapy | 2.130488549 |
| Preoperative lymphocyte | 1.32437106 |
| Preoperative AFP | 1.147654728 |
| Preoperative CA125 | 1.356453066 |
| Preoperative CA72-4 | 1.288799995 |
| Postoperative hemoglobin | 1.37490262 |
| Postoperative neutrophil to lymphocyte ratio | 1.150591242 |
| Postoperative platelet to lymphocyte ratio | 1.395327743 |
| Radscore | 2.786234738 |
